# Distinct cholesterogenic and lipidogenic gene expression patterns in ovarian cancer - a new pool of biomarkers

**DOI:** 10.18632/genesandcancer.87

**Published:** 2015-11

**Authors:** Georgios Pampalakis, Angeliki-Louiza Politi, Anastasios Papanastasiou, Georgia Sotiropoulou

**Affiliations:** ^1^ Department of Pharmacy, School of Health Sciences, University of Patras, Rion-Patras, Greece

**Keywords:** ovarian cancer, biomarker, cholesterol homeostasis, lipid homeostasis

## Abstract

Cancer cells display different metabolic requirements compared to nonmalignant cells imposed by their need for rapid proliferation. Alterations in cellular metabolic pathways of lipid and cholesterol synthesis have been linked to tumorigenesis and cancer progression but have not been exploited in clinical diagnosis. Here, the expression of genes related to cholesterol/lipid metabolism was measured with semiquantitative and real-time RT-PCR in RNA isolated from normal, benign and cancer ovarian tissues. We found that both *SREBF2* and its target gene *DHCR7* are downregulated in ovarian cancer tissues. On the contrary, *SREBF1c* and its target *SCD1* were upregulated. The steroidogenesis regulator *PDE8B* was found downregulated. Oncomine analysis supported these findings, and further revealed that in ovarian cancers, the *SREBF1*-regulated lipidogenic pathway is activated while the *SREBF2*-regulated cholesterogenic pathway is repressed based on expression profiles of *HMGCR* and *DHCR7*. In conclusion, we show that ovarian cancer cells display distinct lipidogenic and cholesterogenic gene expression profiles with potential applications in the development of new biomarkers and/or treatment of ovarian cancer. Reduced cholesterol and enhanced lipid synthesis and *SCD1* expression may provide an explanation for the previously reported increased membrane fluidity of ovarian cancer cells, a finding that merits further investigation.

## INTRODUCTION

Ovarian cancer is a neoplastic growth originating from various parts of the ovary, although most ovarian cancers derive from ovarian surface epithelial cells. Since ovarian cancer constitutes a main cause of mortality in women, understanding its pathophysiology and translation into novel therapeutics is a major concern [1]. Considering that malignant cell proliferation highly depends on nutrients, energy and synthetic activity ensuring duplication of all macromolecules during cell division, it is reasonable that metabolic activities in cycling cells, like cancer cells, are fundamentally different than in non-proliferating cells [2].

Recent studies highlighted that tumors display distinct metabolic programs, and altered cholesterol/ lipid metabolism is emerging as an important process in cancer [3-7]. Clendening et al. [7] showed that *HMGCR* (3-hydroxy-3-methylglutaryl-CoA reductase) and other genes encoding enzymes or regulatory proteins of the mevalonate (MVA) pathway exhibit aberrantly high expression in breast cancers and overexpression of HMGCR or its novel transcript variant lacking exon 13 promoted cellular transformation indicating that HMGCR represents a metabolic oncogene [7]. In breast cancers, upregulation of genes involved in the MVA pathway is directly related to mutant p53 that in turn, interacts with SREBPs to induce expression of cholesterogenic/lipogenic genes [5]. In addition, alteration of lipid metabolism is increasingly recognized as a hallmark of cancer [6]. Indeed, it has been shown that specific pathways are shared between atherosclerosis and cancer pointing to a common transcriptional program [6]. Co-culturing ovarian cancer cells with adipocytes induced lipolysis in adipocytes and transport of lipids to cancer cells that exhibited increased *β*-oxidation. This may provide an explanation for the observed omental metastasis of ovarian cancer [8]. Moreover, ectopic expression of *SREBF1* in MCF10A non-tumorigenic breast cancer cells enhanced lipogenesis in stem-like cells and promoted cell growth and mammosphere formation [4].

To our knowledge no study has been performed to investigate the expression of genes encoding the cholesterogenic and lipidogenic enzymes and the expression of their master regulators *SREBF1* and *SREBF2* in ovarian cancer or any other type of cancer. To examine whether lipid and cholesterol synthesis pathways are altered in ovarian cancer, we analyzed the expression of *SREBF1c*, *SREBF2*, *SCD1*, *DHCR7*, *LDLR* and *PDE8B* in a pilot study. *SREBF1c* is the isoform transcribed from the *SREBF1* gene encoding the transcription factor mainly responsible for the expression of lipidogenic genes, while *SREBF2* encodes the transcription factor of the genes encoding the enzyme of the MVA pathway including the *DHCR7*, that catalyzes the production of cholesterol from 7-dehydrocholesterol and is the causative gene for Smith-Lemli-Opitz syndrome (SLOS, OMIM #270400) [9]. SCD1 is the rate-limiting step of monounsatured fatty acid synthesis [10]. PDE8B is a cAMP-specific phosphodiesterase that controls steroidogenesis, a cholesterol biotransformation process [11]. LDLR is the major protein involved in hypercholesterolemia [12]. We enriched our expression pattern analysis by analyzing microarray data with oncomine and incorporating the expression levels of *HMGCR*, *INSIG1*, *LDLRAP1* and *FASN*. Our data showed for the first time that the *SREBF1* and the *SREBF2* regulated pathways are altered in ovarian cancer with the former exhibiting up-regulation and the latter down-regulation.

## RESULTS

**Figure 1:**
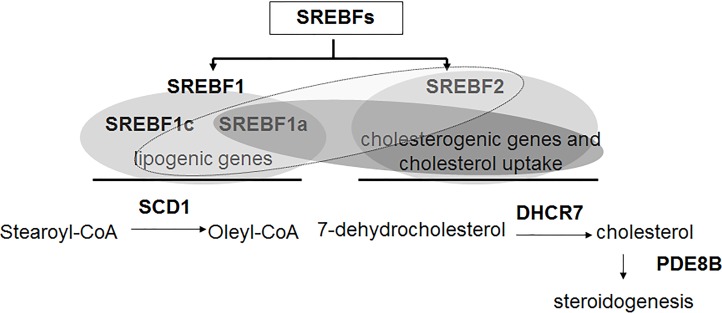
Schematic representation of relative transcriptional activities of SREBFs in association with cholestrerogenic/lipogenic target genes *SREBF* genes comprise *SREBF1* (that encodes the alternative transcripts *SREBF1c* and *SREBF1a*) and *SREBF2*. *SREBF1c* mainly controls the expression of lipogenic genes, *SREBF1a* can activate both lipogenic and cholesterogenic genes and *SREBF2* activates mainly the cholesterogenic genes and lipogenic genes with lower affinity [This figure is a modification of a previously published figure [30]].

**Figure 2:**
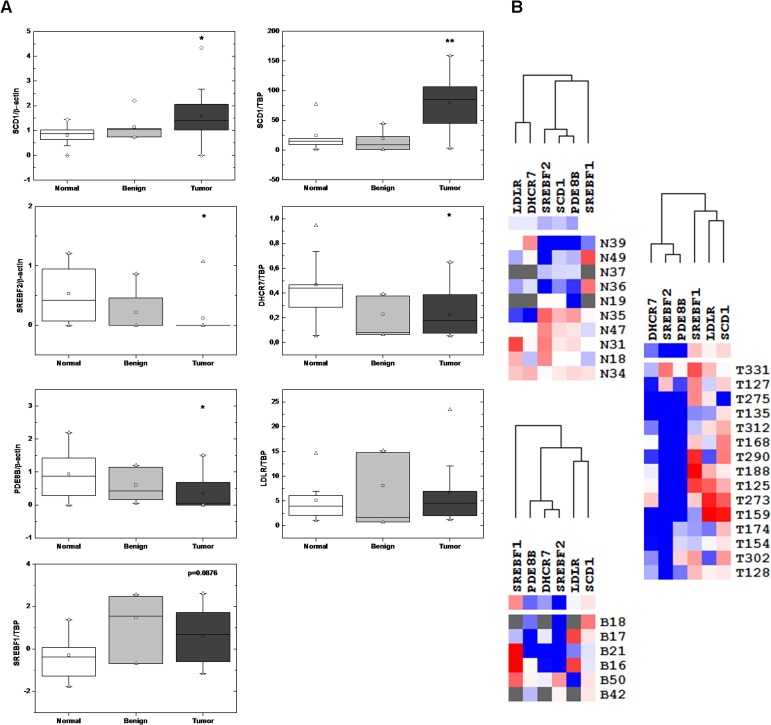
Analysis of gene expression *A,* Box plot diagrams of gene expression. Squares indicate mean values, triangles the 1% and 99% percentile and box limits 25% and 75% over median, respectively. Error bars extend to 10% (low) and 90% (high). Horizontal lines indicate either lower or higher values.*p<0.05, **p<0.005. *B,* Heatmap of gene expression. Red, increased expression; blue decreased expression; white, no change; grey, no available data. N, normal; B benign; T, tumor.

For analysis of gene expression we used 10 normal ovary tissue specimens (average age 52.7 ± 5.7 years, median 51 years), 6 benign (average age 46.3 ± 12.4 years, median 44.5) and 15 ovarian cancer specimens (average 56.6 ± 13.9, median 57). Figure 1 depicts the genes that were chosen for expression level analysis. *PDE8B* is the only gene from our list that is not regulated by SREBPs. As shown in Figure 2A, 2B and in Table 1, up-regulation of *SCD1* expression was clearly observed in ovarian cancer compared to normal and benign tissues. Contrary, *SREBF2* and its target *DHCR7* as well as *PDE8B* were found downregulated. Expression of *LDLR* was statistically not significantly different between normal and cancer tissues (p=0.584, t-test). *SREBF1c* was up-regulated in cancer samples compared to normal samples (Figure 2A, and B, Table 1) although the p value was slightly larger than 0.05 (0.0878, t-test, and 0.0894, Fisher exact test) which may be due to a rather low number of analyzed samples. Up-regulation of *SREBF1c* is in accordance with the up-regulation of its target *SCD1*. Indeed, hierarchical clustering indicated that *SREBF2* and *DHCR7* are clustered together in cancer cells as well as *SREBF1* and *SCD1*. Interestingly, *PDE8B* clusters together with *SREBF2* and *DHCR7*, although *PDE8B* has not been described to be associated with the MVA pathway or controlled by *SREBFs*.

**Table 1:** Differential gene expression in normal and cancer ovarian tissues Analysis of experimental data was carried out according to the Fischer exact test

Gene	Method	Cutoff*	High expression#	Low expression#	*P*
SCD1	RT-PCR	1.04	Normal: 2 Cancer: 11	Normal: 8 Cancer: 4	0.0154
	RT-qPCR	30.00	Normal: 2 Cancer: 14	Normal: 6 Cancer: 1	0.0017
DHCR7	RT-qPCR	0.27	Normal: 7 Cancer: 4	Normal: 1 Cancer: 11	0.0094
PDE8B	RT-PCR	0.85	Normal: 6 Cancer: 2	Normal: 4 Cancer: 13	0.0280
SREBF2	RT-PCR	0.07	Normal: 8 Cancer: 3	Normal: 2 Cancer: 12	0.0051
SREBF1c	RT-qPCR	0.40	Normal: 2 Cancer: 10	Normal: 6 Cancer: 5	0.0894

* Cutoff values were arbitrarily set and correspond to ratio of gene of interest expression against the reference gene expression.

# High/low expression indicates values higher or lower than the cutoff.

We could not find any correlation between gene expression with either stage or grade of cancer. For the panel of tissue specimens analyzed here, distribution of gene expression levels did not show correlation with disease stage or grade. For example, PDE8B gene expression with the cutoff value shown in Table 1, 7/8 cancer samples were below cutoff for stage III and 5/6 for stages I+II (p=1.000) (one cancer sample for which grade and stage were not known was excluded from the statistical analysis). According to grade, 7/8 cancer samples with grade III were below the cutoff and 5/6 for grades I+II. The same results were obtained for the other genes (data not shown).

**Figure 3:**
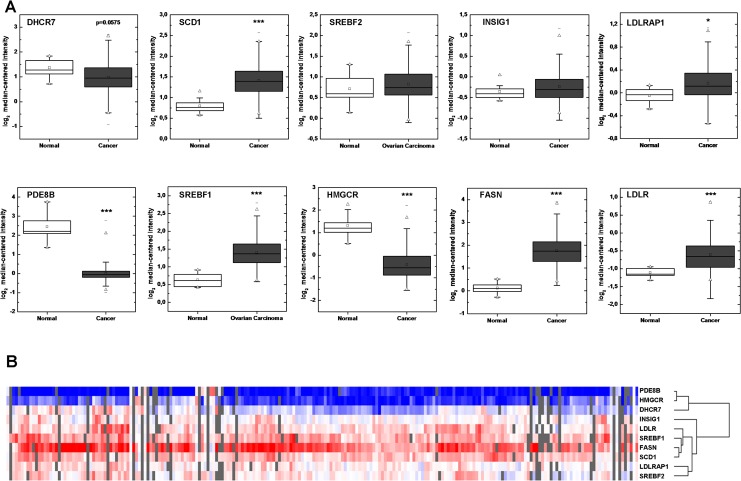
Oncomine analysis To prove the validity of our findings we analyzed publically available microarray data from [13]. Total number of samples: normal 10, ovarian carcinoma 185, *p<0.05, ***p<0.0005. Data are presented as box plot expression of individual genes (A) or as heatmap (B). Heatmap colors are as in Figure 2.

Oncomine was applied to analyze the well-established microarray datasets of [13] encompassing 10 normal and 185 ovarian cancer tissue samples. We have enriched the analysis by incorporating additional genes including *LDLRAP1*, *LDL*, *FASN*, *HMGCR* and *INSIG1*. Our results shown in Figure 2A are in accordance with the microarray data (Figure 3A) with the exception of *SREBF2* for which no correlation could be found in the Bonome dataset (p=0.854) and *LDLR*, which was found up-regulated in ovarian carcinomas. LDLRAP1 that is necessary for the uptake of cholesterol by LDLR [12] was also found up-regulated in ovarian carcinomas. Up-regulation of *SREBF1* was confirmed in the microarray data and was coordinated with increased *FASN* and *SCD1* expression, the later validated both in our experimental set and in the microarray data. Microarray data analysis confirmed that *HMGCR* is strongly repressed in ovarian carcinomas, while induction of *SREBF1* is in accordance with our experimental data showing increased *SREBF1c* in ovarian clinical cancer specimens. Our integrated data point to suppression of the MVA pathway and coordinated activation of the lipogenic pathway in ovarian cancer. Finally, *INSIG1* that is regulated by both SREBFs [14] was not found deregulated in ovarian cancer compared to normal samples.

A heatmap generated by Treeview depicts the profile of clinical samples (Figure 3B). It clusters together genes with common expression profiles and confirms that the expression of *SREBF1*, *SCD1* and *FASN* is coordinately up-regulated in ovarian cancers. Moreover, *PDE8B* clustered together with *HMGCR* and *DHCR7* indicating that its altered expression (down-regulation) is correlated with the expression of MVA pathway genes.

## DISCUSSION

Whereas alterations to metabolism of glucose, amino acids, and fatty acids have been extensively studied in cancer [15], cholesterol metabolism is a relatively understudied field [16]. Maintenance of cholesterol/lipid homeostasis is fundamental for the growth of eukaryotic cells including mammalian cells. Currently, it has been demonstrated that cancer cells display altered cholesterol/ lipid metabolism [5, 6]. Here, we show that genes involved in cholesterol/lipid biotransformation are altered in ovarian cancers.

We found that suppression of *SREBF2* in ovarian cancers results in down-regulation of cholesterogenic target genes such as the *DHCR7*, while induction of *SREBF1* is associated with increased expression of lipid synthesis genes such as the *SCD1*, which was found highly elevated. In accordance, Nie et al. [17] showed by immunohistochemistry that SREBP1 protein levels are elevated in ovarian cancers. SCD1 has a protective effect in cancer cells that *de novo* synthesize high amounts of saturated fatty acids since saturated fatty acids trigger programmed cell death through a process referred as lipoapoptosis [10]. Indeed, ablation of *SCD1* in lung cancer cells reduces their ability to grow xenografts in immunocompromized mice and *SCD1* overexpression correlates with genetic predisposition for liver cancer in rodents [10]. A recent immunohistochemical study showed that *SCD1* is elevated in breast, prostate, lung, renal, and ovarian cancers [18, 19]. Up-regulated expression of *SCD1* in hepatocellular carcinoma has been associated with resistance to chemotherapy-induced apoptosis [20]. Further, we have found that *PDE8B* is strongly down-regulated in ovarian cancer. As the PDE8B is a negative regulator of steroidogenesis [11], its down-regulation, is expected to drive the synthesis of steroids that are essential in supporting the growth of ovarian cancer cells.

Previously, microarray profiling of ovarian epithelial cells exposed to progesterone detected up-regulated expression of gene transcripts encoding for enzymes of the MVA pathway, LDLR, and FASN [21]. This gene set was suggested to mediate the protective role of progesterone against ovarian cancer development. In support of these findings, our gene expression analysis and the analysis of Bonome microarray data [13] showed strong down-regulation of cholesterogenic genes in ovarian cancer, including *HMGCR*. In contrast, in other types of cancer, MVA pathway was shown to drive cellular transformation. Previous studies involving analysis of tissue microarrays showed that patients with ovarian tumors expressing high levels of HMGCR had a significantly favorable prognosis [22]. Pitfalls of this study include lack of correlation with normal tissue expression and a later study that demonstrated non-specific crossreactivity of the antibody [7].

Aberrant regulation of cholesterol and lipid metabolism may significantly impact on cell membrane fluidity, since their metabolic products are components of cellular membranes. Previously it was observed that in SKOV-3 ovarian adenocarcinoma cells, decrease in membrane fluidity reduces aggressiveness of these cells [23]. Consistently, we found that the cholesterol pathway is repressed in ovarian cancer cells. Reduced cholesterol is likely responsible for decreased membrane fluidity corroborated by increased contents of monounsatured fatty acids produced by elevated *SCD1*. Recently, it has been reported that in breast cancer, cholesterol metabolites can either promote (e.g. 27-hydroxycholesterol) or inhibit tumor formation (e.g. dendrogenin A) [24]. This may also hold true for ovarian cancer and could account for the differential expression of cholesterogenic and lipidogenic genes, a fact that merits further investigation.

Recently, lipid profiles emerged as an alternative approach to stratify breast cancers thus, opening new ways to be exploited in molecular cancer diagnostics [25]. Based on lipid profiles determined by Raman microspectroscopy and multivariate statistical techniques, an algorithm was developed to distinguish breast cancer cells with enhanced metastatic ability and to monitor epithelial-to-mesenchymal transition [25]. However, analysis of lipids is more complex and requires sophisticated instrumentation as opposed to mRNA quantification, making its application for routine monitoring rather difficult.

In summary, this is the first report describing altered expression profiles of key genes involved in cholesterol and lipid biosynthesis/biotransformation in ovarian cancer. These expression profiles reveal that lipidogenic and cholesterogenic pathways are differentially regulated in ovarian cancer impacting on the metabolome and hence the biology of ovarian cancer cells. The currently accepted general concept is that cholesterol and sterols synthesis has evolved symbiotically with fatty acid synthesis in order to form structural components of cell membranes (they both require the same carbon acetyl-CoA and hydrogen NADPH sources, and are under the control of SREBPs) [26]. However, our data indicate that under certain conditions (e.g. ovarian cancer) there are ways to overcome this concerted regulation and instead differentially regulate one pathway relative to the other. Further, our results may provide novel targets for pharmacological intervention. Towards this direction, it was demonstrated that targeting SCD1 for inhibition increases apoptosis of renal carcinoma cells *in vitro* and *in vivo* [19] and lung cancer cells *in vivo* [27], while in ovarian cancer, FASN inhibitors induce cell cycle blockage and stimulate apoptosis. Thus, *FASN* is considered a metabolic marker of cell proliferation [28].

## MATERIALS AND METHODS

### Patients and clinical specimens

Tissue specimens were collected by Dr D Katsaros at the University of Turin, Italy and have been described previously [29]. All tissues were obtained after patients' written consent under a general tissue collection protocol approved by the Institutional Review Board and the University of Turin. Samples were snap-frozen in liquid N_2_ and then stored at −80°C until use.

### RNA extraction and reverse transcription

Total RNA extraction was carried out with RNeasy (Qiagen) and RNAs were treated with DNase according to manufacturer's instructions. RNA integrity was confirmed by agarose gel electrophoresis. 500 ng of RNA were reverse-transcribed with Superscript II (Invitrogen) using an oligo-dT primer and the cDNAs were recovered in 21 μl.

### Semiquantitative polymerase chain reaction

*Taq* DNA polymerase with ThermoPol Buffer (NEB) was used to amplify *β*-actin, *SCD1*, *SREBF2*, and *PDE8B* partial gene sequences. Primers, annealing temperatures and cycle numbers were as follows: *β*-actin, Forward: ACA ATG AGC TGC GTG TGG CT, Reverse: TCT CCT TAA TGT CAC GCA CGA (62oC, 35 cycles); *SCD1*, Forward: TGC AGG ACG ATA TCT CTA GC, Reverse: ACG ATG AGC TCC TGC TGT TA (51oC, 40 cycles); *SREBF2*, Forward: AAG TCT GGC GTT CTG AGG AA, Reverse: CAC AAA GAC GCT CAG GAC AA (55oC, 40 cycles); *PDE8B*, Forward: CAG AAT CGT CGC TAT CCG TCC A, Reverse: ACC TTT AAG CCC AGA TAA ACC A (50oC, 40 cycles). PCR products were resolved in 2% agarose and visualized with ethidium bromide. Quantification of PCR products was performed using ImageJ (http://rsb.info.nih.gov/ij/download.html). Data are reported as intensity (gene of interest) / intensity (*β*-actin).

### Quantitative RT-PCR

50 ng of total RNA were used in one-step RT-qPCR. The reaction was carried out in 25 μl with Kapa SYBR Fast One-Step qRT-PCR (Kapa Biosystems) in a Rotor Gene 3000 (Cobert Research). The following conditions were applied: 5 min at 42oC for reverse transcription, 94oC for 5 min followed by 45 cycles (40 for TBP, TATA-binding protein) of 94oC for 15 sec, 54oC for 20 sec (30 sec for TBP), and 72oC for 20 sec (30 sec for TBP). Primers for *SCD1*, *LDLR*, *DHCR7* and *SREBF1c* amplification were as follows: *SCD1*, Forward: CCG GGA GAA TAT CCT GGT TT, Reverse: GCG GTA CTC ACT GGC AGA GT; *LDLR*, Forward: GGC AAC CGG AAG ACC ATC TTG GA, Reverse: CGG TTG GCA CTG AAA ATG GCT TC; *DHCR7*, Forward: CCC AGC TCT ATA CCT TGT GG, Reverse: CCA GAG CAG GTG CGT GAG GAG; *SREBF1c*, Forward, GGA GGG GTA GGG CCA ACG GCC T, Reverse GGC CAG GGA AGT CAC TGT CTT G. The gene encoding TBP was used for RT-qPCR normalization. Primers for TBP amplification were obtained from Qiagen (QuantiTect Primer Assay). All samples were analyzed in triplicates.

### Bioinformatic analysis

Microarray data were retrieved from Oncomine and statistically analyzed with Origin 8.0. Further, the microarray gene expression data were normalized against the median of normal gene expression and used for hierarchical clustering. Hierarchical clustering was performed with Cluster and visualization of results with TreeView (http://rana.lbl.gov/EisenSoftware.htm). Hierarchical clustering was also performed in the experimental dataset. For this, expression levels from clinical specimens were converted to log_2_ and normalized against the mean of normal expression.

### Statistical analysis

Differences between groups were analyzed with two-sample t-test (Origin 8.0) or with Fisher exact test (http://graphpad.com/quickcalcs/contingency1.cfm).

### Sequencing

Representative PCR products were gel purified with Nucleospin Gel and PCR Clean-up (Macherey-Nagel) and directly sequenced with the sense PCR primer (VBC Biotech, Austria) in order to confirm the identity of the products.

## Abbreviations

DHCR7: 7-dehydrocholesterol reductase
FASN: fatty acid synthase
HMGCR: 3-hydroxy-3-methyl-glutaryl-coenzyme A reductase
LDLR: low density lipoprotein receptor
MVA: mevalonate pathway
PDE8B: phosphodiesterase 8B
SCD1: stearoyl-CoA desaturase-1
SREBF: sterol regulatory element binding transcription factor (gene)
SREBP: the protein encoded by SREBF
TBP: TATA-binding protein
